# Ethiopia has a long way to go meeting adolescent and youth sexual reproductive health needs

**DOI:** 10.1186/s12978-022-01445-3

**Published:** 2022-06-13

**Authors:** Teshome W. Admassu, Yordanos T. Wolde, Mirgissa Kaba

**Affiliations:** 1 LHD International Consult LLC, Silver Spring, MD US; 2 Talent Youth Association, Addis Ababa, Ethiopia; 3 Department of Preventive Medicine School of Public Health, College of Health Sciences, Addis Ababa University, Addis Ababa, Ethiopia

**Keywords:** Adolescent, Youth, Sexual and reproductive health needs, Adolescents and youth-related interventions, Ethiopia

## Abstract

Ethiopia has the second-largest youth population in Africa with about 37.4 million people aged 10–24 years. To meet the needs of this population group, adolescent, and youth health (AYH) programs, including those focused on sexual and reproductive health (SRH) and youth development, have gained traction in Ethiopia in the last two decades, especially following the 2005 election in Ethiopia. However, adolescents and youths in Ethiopia continue to face a high burden of morbidity and mortality from multiple factors including, teenage pregnancy, unplanned pregnancy, compromised nutrition, HIV and STIs, unsafe abortion, early and child marriage, and unmet needs for family planning. To date, adolescents and youth-related interventions in Ethiopia are fragmented under various ministries, uncoordinated, underfunded, project-oriented, lack effective policy implementation, and lack meaningful participation of young people. Addressing adolescents and youth health and development issues require evidence-based, well-tailored, at scale, intensified, coordinated, and holistic national responses. Therefore, there is a need to advocate for a realization of robust government commitment to ensure a multi-sectoral, coordinated, at scale, and well-funded national response to address the multifaceted needs of young people in Ethiopia including their sexual and reproductive health. An example to emulate is the HIV/AIDS response in Ethiopia, which was led by a national council chaired by the President of the Federal Democratic Republic of Ethiopia and spearheaded by a secretariat leveraging resources and implementing a multisectoral national plan down to the kebele level.

## Main text

More than one of every four Ethiopians is an adolescent 10–19 years of age while one in three is a young person aged 10–24 years [1]. Adolescents and youths 10 to 29 years are estimated to constitute 42% of the total population [2]. This section of the population should, in principle, be targeted with the right investment to contribute to the development of the country [3]. The investment among others includes enacting, designing, and implementing policies and programs that foster and support robust national youth development programs.

To meet the needs of this population group, adolescent, and youth development interventions, including those focused on health, and sexual and reproductive health (SRH), have gained traction in Ethiopia during the last two decades, especially following the 2005 election in Ethiopia [4].

The youth development policy environment in Ethiopia is favorable. The government established a youth dedicated ministry, enacted various policies and laws, developed national strategies, frameworks, guidelines, and documents to strategically guide youth development efforts in the country. Though, the government’s position regarding young people has dramatically shifted since the enactment of the 2004 National Youth Policy (NYP) [5], which recognized youths as change agents and sought to ensure ownership and involvement of the youth in the country’s development initiatives and contradicted the sentiment of the Vagrancy Control Proclamation, made that same year that considered young people as problems [6].

Traditionally, youth-related advocacy efforts in Ethiopia have been driven by NGOs, donors, and community-based organizations, and included youth-led initiatives. These advocacy efforts have led to notable gains including the establishment of the Ministry of Youth, Sport, and Culture, in 2001 and the enactment and development of various, policies, laws, strategies, and national documents to improve the status of young people in Ethiopia. The revised family code in 2000 [7] and the revised criminal code of Ethiopia in 2004 [8] that has liberalized abortion in Ethiopia; Development Package for Urban Youth of 2006 [9] and the Ethiopian Youth Development and Transformation Package of 2017 [10] contributed to efforts underway to adolescent and youth development. The revised family code, for instance, has saved thousands of adolescents from child marriage by instituting a minimum age of marriage [11]; the abortion law avoided a significant number of maternal deaths by expanding the provision of comprehensive abortion care services to adolescent and young girls [12, 13]. Other strategies include the 2007 national adolescent and youth reproductive health (AYRH) strategy [14], and the more comprehensive national adolescent and youth health (AYH) strategy of 2016 [15]. In general, these policies, laws, strategies, and packages, provided strategic guidance to improve the well-being of youths, girls, and young women.

Nonetheless, adolescents and youths in Ethiopia continued to suffer from a high burden of morbidity and mortality associated with reproductive health consequences [16]. Ethiopia has the fourth-highest absolute number of women globally married or in a union before the age of 18 years [17]. Over 76% of girls aged 15–19 and 65% of young women aged 20–24 who are married/in union are not using any method of contraceptives. The unmet need for family planning among married girls aged 15–19 years and 20–24-year-olds is 32.8% and 22.4% respectively [18]. Close to eight out of ten adolescents seeking legal abortion or postabortion care were not using family planning methods at the time of their pregnancy [13]. In Ethiopia still. Child marriage below the age of 18 years is not an exception. About 22.2% of women aged 20–24 years gave birth before they reached age 18 years [18]. Nearly half (47%) of adolescent girls aged 15–19 have undergone female genital mutilation [1]. Nearly two-thirds (64%) of women and girls aged 15–24 years believe wife-beating can be justified [13].

As part of its Family Planning 2020 commitments, the government of Ethiopia set ambitious goals to meet the reproductive health needs of adolescents from what these were in 2017. The commitment includes increasing modern contraceptive prevalence (mCPR) among adolescent girls aged 15–19 years from 32 to 40% and 20% to 10% among young women aged 20–24 years; and reducing the unmet need for family planning for those 15–19 and 20–24 years of from age from 20–10% and 18–10% and reducing adolescent pregnancy from 12 to 3%; respectively by the 2020 target [19].

Despite the efforts and progress by the government and its development partners yet there is a long way to meet the targets and ensure adolescent and youth sexual reproductive health. Currently, teenage pregnancy, unplanned pregnancy, HIV and STIs, unsafe abortion, early and child marriage, and unmet needs for family planning and contraceptive, risky sexual practice, among others, are major concerns that require well-tailored, at scale, intensified, coordinated, and holistic national responses [2, 4, 13, 17].

As pointed out above, the policy environment is conducive. Yet, several challenges have prevented the full implementation of policies and programs that meet the sexual and reproductive health needs of youths. Continual restructuring of the Ministry dedicated to youth issues gravely hinders the implementation of the NYP and attainment of its goals. To date, there is no national AYH and/or youth development program and ongoing efforts lack sufficient resource allocation by the government. Effectively addressing youth issues is further hampered by poor implementation of the NYP, poor coordination among various ministries and key stakeholders; lack of age and sex-disaggregated data across ministries, and lack of meaningful engagement of young people at all levels to address issues that affect their lives [4].

As stipulated in the NYP, it is required to establish the youth councils, inter-federal government offices committee and inter-regional bureaus committee, a consortium of non-governmental bodies, and Nationwide Youth Forum to meet provisions in the NYP [4, 5]. This remains yet outstanding affecting adolescent and youth SRH programming and youth development efforts at large. Furthermore, adolescent and youth SRH programming by different stakeholders are in silos, uncoordinated, and unsynchronized as the Ministry of Women, Children and Youth^1^ lacks the mechanism to effectively coordinate and implement youth-related responses with other ministries and stakeholders.

Regional health and youth bureaus are ill-equipped to design, implement, monitor, and evaluate tailored and evidence-based strategies, approaches, and interventions. For instance, there is no regional health or youth bureau that developed a regional policy implementation strategy and or intervention to date. Moreover, existing national and regional interventions, which are mainly funded by development partners, are short-lived projects implemented in piecemeal and skewed towards urban settings [20]. Most-AYSRH focused interventions are funded by international NGOs as projects, and often at a small scale, with no clear direction and/or mechanisms to scale up and sustain successful projects [20, 21]. The engagement of young people in contributing to their own wellbeing and development is weak due to poor government commitment and the absence and implementation of national youth engagement strategies. These youth engagement strategies would allow young people, who have a stake in this programming, to contribute to planning, implementation, and follow-up of projects targeting them [22].

Education, especially comprehensive sexual education (CSE), has positive effects including increasing young people’s knowledge and improving their attitudes related to sexual and reproductive health and behaviors as they move into adulthood. The government of Ethiopia has not yet fully implemented its Eastern and Southern Africa (ESA) commitment endorsed in 2013 to improve the sexual and reproductive health rights of young people by increasing coverage and access to age-appropriate, evidence-based, inclusive CSE and friendly SRH services for both boys and girls [22]. Despite the government’s commitment, there is no national CSE program in the country to date.

Therefore, there is a strong need to advocate for a realization of robust government commitment to ensure a multi-sectoral, coordinated, at scale, and well-funded national response to address the multifaceted needs of young people in Ethiopia including their sexual and reproductive health. A useful model to emulate can be found in Ethiopia’s HIV/AIDS response, which was led by a national AIDS council (NAC) that was established in 2000 and chaired by the President of Ethiopia to oversee the implementation of the federal and regional HIV/AIDS plans, examines, and approves annual plans and budgets and monitors plan performance and impact. The council established a secretariat office to lead, coordinate, and mobilize resources for the implementation of the multi sectoral national HIV/AIDS strategy and plan along with the Federal structure. The NAC spearheaded the multi-sectoral forum composed of government, private, non-governmental, religious, and civic society representatives including youth initiatives and people living with HIV/AIDS, through its secretariat, HIV/AIDS prevention, and control office (HAPCO) [23].

It is time for the government to establish such a high-level body led by the country’s top leadership to guide adolescent and youth development programs and ensure coordination among diverse stakeholders and resource allocation. This may call for reviewing existing policies to address current issues of young people, improving coordination efforts and interventions by various ministries and stakeholders, leveraging resources for youth development programs, building the capacity of youth-led initiatives, allocating funding to youth interventions, particularly for youth and youth-led organizations, and generating and documenting evidence on the effectiveness of interventions.

## Conclusion

The youth development environment in Ethiopia has made great progress in addressing the multifaced needs of young people, yet critical steps are still needed to fully engage young people and to ensure that their needs and rights are met. The positive development in this arena is the result of continued advocacy efforts by various stakeholders including NGOs, donors, and civil society organizations, including youth-led and youth-serving organizations.

There is a strong need to advocate for a robust government commitment led by the country’s top leadership to provide strategic guidance, allocate national funding and improve coordination mechanisms following good lessons from the coordination of HIV/AIDS response through NAC that made a well-coordinated multisectoral response possible.

Finally, the well organized and evidence-informed advocacy effort should gear up towards revisions of outdated policies, strategies, approaches, packages, and frameworks to address holistic adolescent and youth development including adolescent and youth sexual and reproductive health.

## Data Availability

Not applicable.

## Abbreviations

AIDS: Acquired immunodeficiency syndrome
AYH: Adolescent and youth health
AYRH: Adolescent and youth reproductive health
AYSRH: Adolescent and youth sexual and reproductive health
CSE: Comprehensive sexual education
ESA: Eastern and Southern Africa
HAPCO: HIV/AIDS prevention and control office
HIV: Human immunodeficiency virus
mCPR: Modern contraceptive prevalence
NAC: National AIDS Council
NGOs: Non-Governmental Organizations
NYP: National youth policy
SRH: Sexual and reproductive health
STIs: Sexually transmitted infections

## Footnotes

1 The Ministry of Women, Children, and Youth is the current name of the ministry previously known as the Ministry of Youth, Sport, and Culture.
